# Supplementary material for: SARS-CoV-2 ORF6 disrupts nucleocytoplasmic trafficking to advance viral replication
Source: Commun Biol. 2022 May 19;5:483. doi: 10.1038/s42003-022-03427-4 (PMC9120032; doi:10.1038/s42003-022-03427-4)

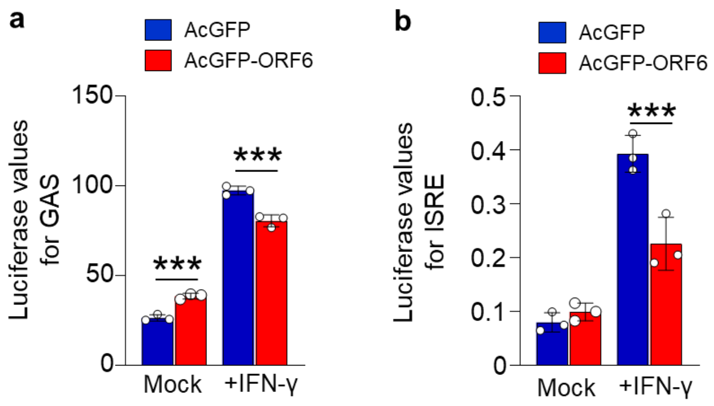


**Supplementary Figure 1. Raw luciferase values in ORF6-transfected cells following IFN-γ stimulation**

**a-b)** Raw luciferase values for the GAS-Luc (**a**) or the ISRE-luc (**b**) in AcGFP or AcGFP-ORF6-transfected Huh7 cells following IFN-γ stimulation (n=3). Fig. 1e and f are relative luciferase values based on the graphs **a** and **b**, respectively. ***$P < 0.001$, one-way ANOVA. error bars represent SD.

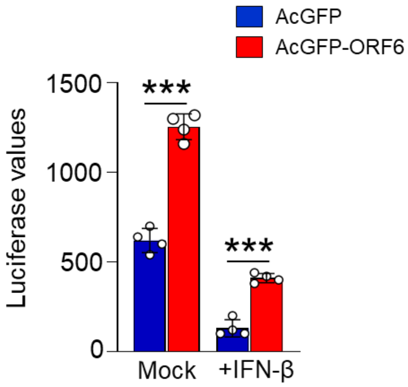


**Supplementary Figure 2. ORF6 suppresses IFN-β-mediated down-regulation of the viral replication**

Vero E6 replicon stable cell lines were transfected with AcGFP or AcGFP-ORF6, and then the cells were treated with IFN-β (final conc. 50 ng/mL) for 32 h. Luciferase values were determined by luciferase assay (n=4). ***P < 0.001, one-way ANOVA. error bars represent SD.

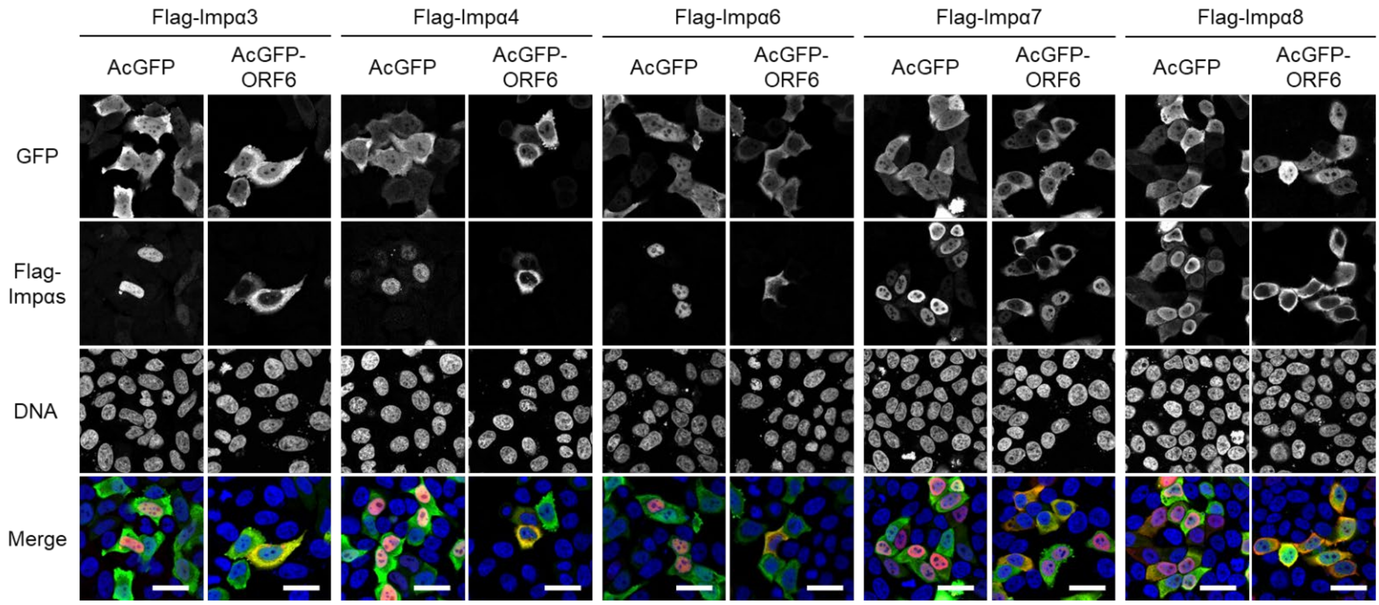


**Supplementary Figure 3. Subcellular distribution of Flag-importin α subtypes**

Subcellular localization of Flag-importin α3, α4, α6, α7, and α8 in HeLa cells expressed with AcGFP or AcGFP-ORF6. The GFP protein (green) and the Flag-importin α proteins (red) were detected using the anti-GFP or anti-Flag antibodies, respectively. DNA was stained with DAPI (blue). Scale bars: 30 µm.

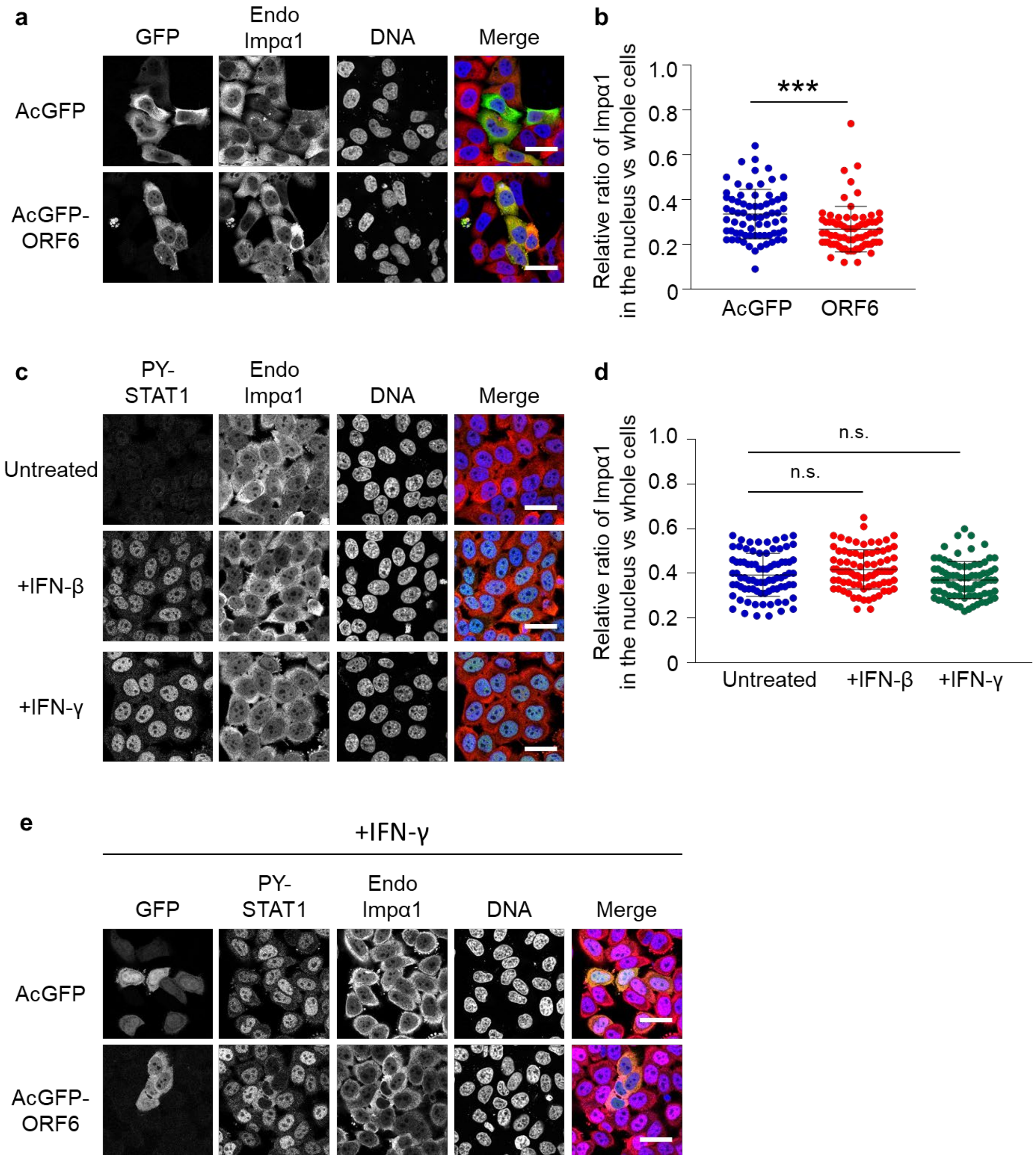


**Supplementary Figure 4. Subcellular distribution of endogenous importin α1 in ORF6-transfected HeLa cells**

**a)** Subcellular localization of endogenous importin α1 (Endo Impα1) in AcGFP or AcGFP-ORF6 HeLa cells. GFP (green) and importin α1 (red) were detected using specific antibodies. DNA was stained with DAPI (blue). Scale bars: 30 μm. **b)** The graph represents relative values of importin α1 in the nucleus against the whole cells in **a**. Signal intensities of total 80 different nuclei from two independent experiments were measured, and statistically analyzed using student's t-test. ***$P<0.001$. error bars represent SD. **c)** Subcellular localization of PY-STAT1 and endogenous importin α1 (endo Impα1) in HeLa cells treated with IFN-β or IFN-γ. PY-STAT1

(green) and importin α1 (red) were detected using the specific antibodies. DNA was stained with DAPI (blue). Scale bars: 30 µm. **d)** The graph represents relative values of the fluorescent of importin α1 in the nucleus against the whole cells in **c**. Signal intensities of total 80 different nuclei from two independent experiments were measured, and statistically analyzed using a one-way ANOVA. n.s.: not significant. error bars represent SD. **e)** Subcellular localization of PY-STAT1 and endogenous Impα1 in AcGFP or AcGFP-ORF6-transfected HeLa cells treated with IFN-γ. Anti-PY-STAT1 (magenta) and anti-importin α1 (red) antibodies were used for detection. DNA was stained with DAPI (blue). Scale bars: 30 µm.

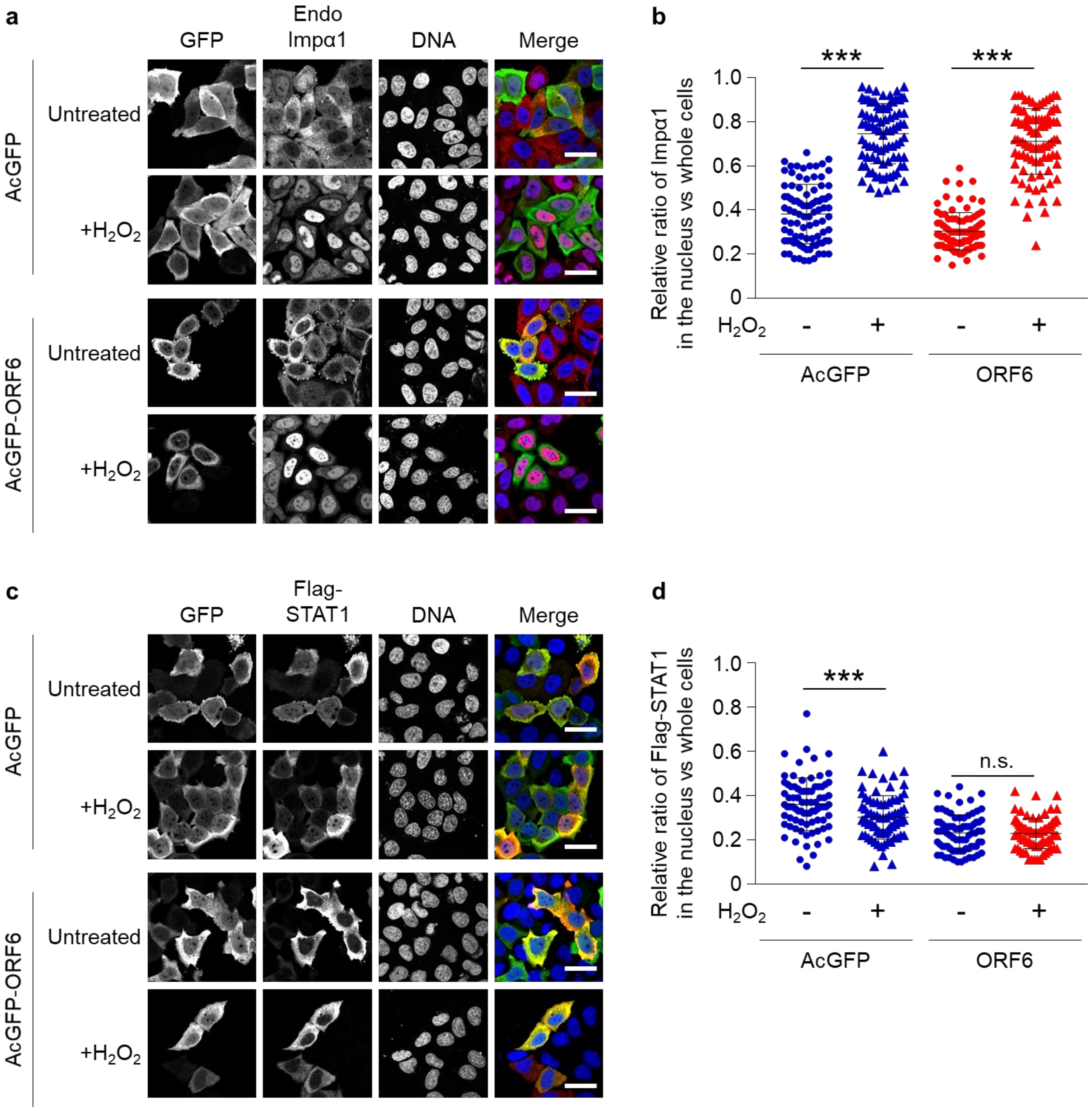


**Supplementary Figure 5. Importin α1 shuttles in ORF6-transfected cells**

**a)** Subcellular localization of endogenous importin α1 (Endo Impα1) in HeLa cells expressed AcGFP or AcGFP-ORF6 following hydrogen peroxide (200 μM $H_2O_2$) treatment. GFP (green) and importin α1 (red) were detected using the anti-GFP or anti-importin α1 antibodies, respectively. DNA was stained with DAPI (blue). Scale bars: 30 μm. **b)** The graph represents the relative values of the fluorescent of importin α1 in the nucleus against the whole cells in **a**. Signal intensities of total 90 different nuclei from two independent experiments were measured, and statistically analyzed using a one-way ANOVA. ***$P<0.001$. error bars represent SD. **c)** Subcellular localization of Flag-STAT1 in HeLa cells transfected AcGFP or AcGFP-ORF6 following hydrogen peroxide (200 μM $H_2O_2$) treatment. GFP and Flag-STAT1 were detected using anti-GFP (green) or anti-Flag (red)

antibodies. DNA was stained with DAPI (blue). Scale bars: 30 μm. **d)** The graph represents the relative values of Flag-STAT1 in the nucleus against the whole cells in **c**. Signal intensities of total 80 different nuclei from two independent experiments were measured, and statistically analyzed using a one-way ANOVA. ***$P<0.001$, n.s.: not significant. error bars represent SD.

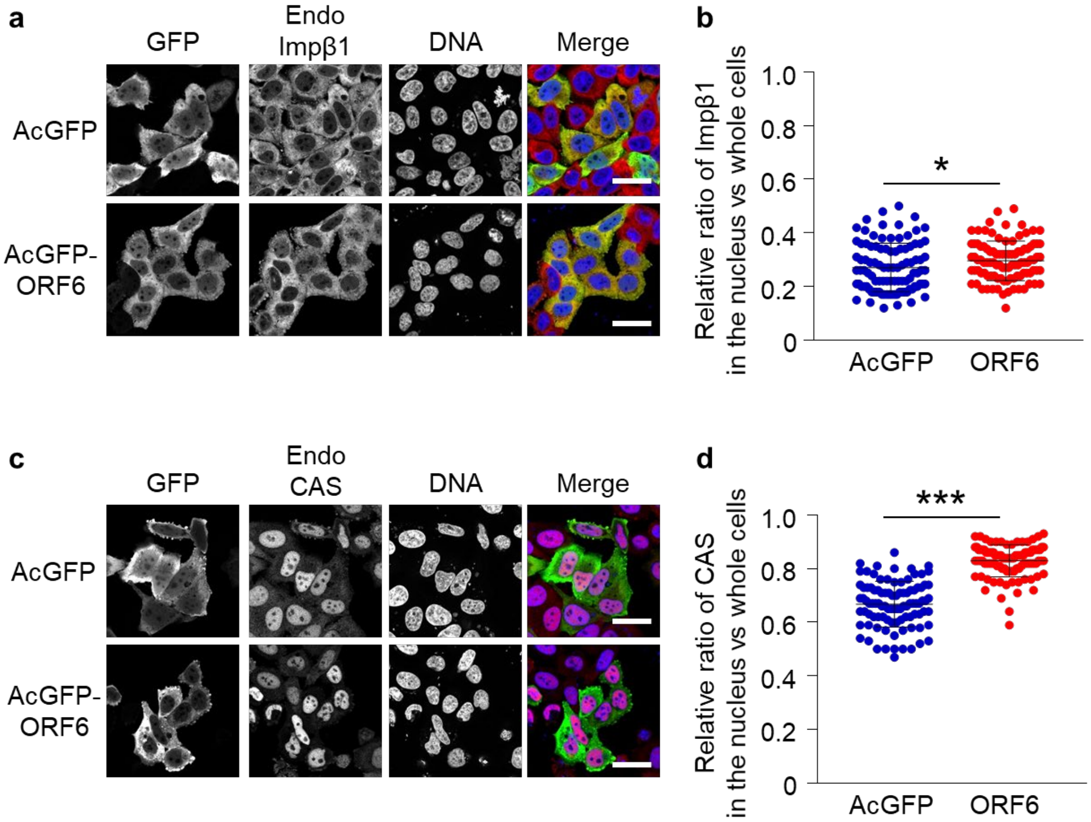


**Supplementary Figure 6. Subcellular distribution of endogenous importin β1 or CAS in ORF6-transfected HeLa cells**

**a-d)** Subcellular localization of endogenous importin β1 (Impβ1; **a**) or endogenous CAS (**c**) in AcGFP or AcGFP-ORF6 HeLa cells. GFP (green) and importin β1 (red) or CAS (red) were detected using specific antibodies. DNA was stained with DAPI (blue). Scale bars: 30 μm. The graph represents the relative values of the fluorescent of importin β1 (**b**) or CAS (**d**) in the nucleus against the whole cells. Signal intensities of total 70-100 different nuclei from two independent experiments were measured, and statistically analyzed using student's t-test. *P<0.05, ***P<0.001. error bars represent SD.

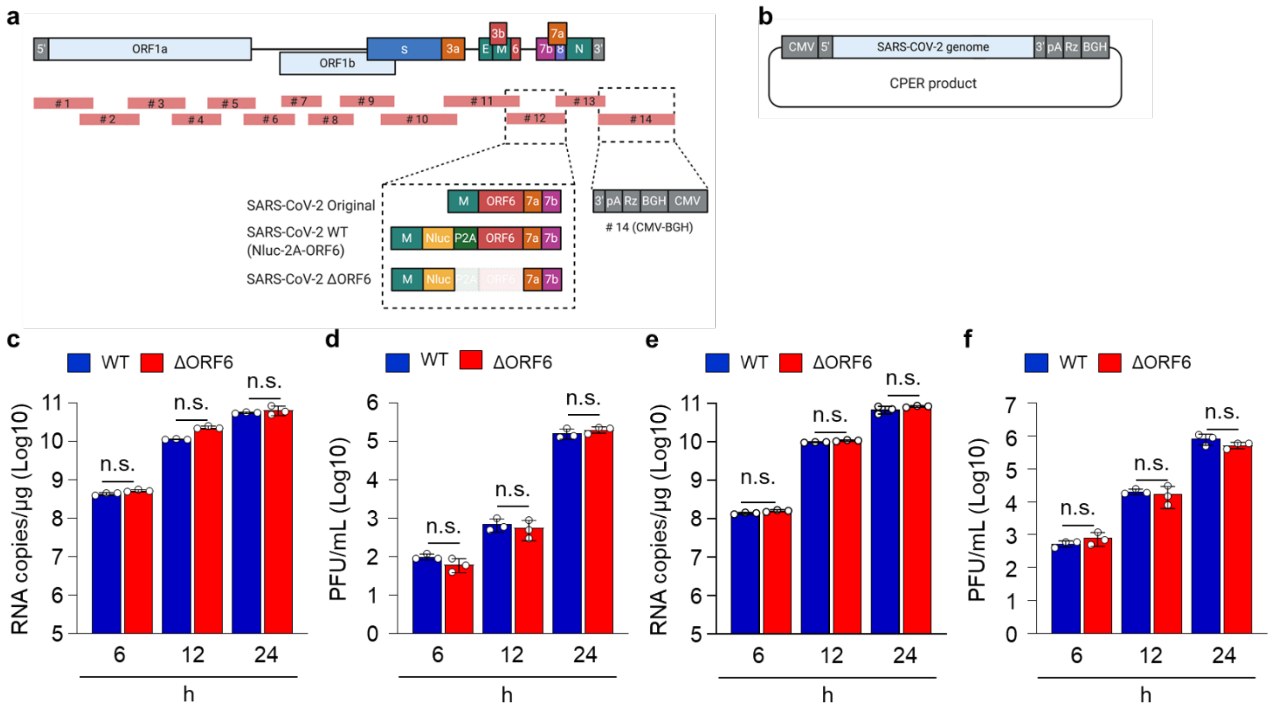


**Supplementary Figure 7. Establishment of recombinant SARS-CoV-2 by the circular polymerase extension reaction**

**a)** Schematic representation of recombinant SARS-CoV-2. A total of 13 fragments (#1-#13) that cover the viral genome were amplified with 40-60-nt overlapping ends. The fragments were mixed with the #14 fragment, which contained the CMV promoter (CMV) followed by the first 20 nt of SARS-CoV-2 genome and, at the other end, the 3' UTR of SARS-CoV-2, a synthetic poly(A) tail (pA), hepatitis delta virus ribozyme (Rz), and bovine growth hormone polyadenylation sequence (BGH). NLuc, NanoLuc gene; P2A, Porcine teschovirus 2A peptide. **b)** The genetic structure of the CPER product of the recombinant SARS-CoV-2. A total of 14 fragments were assembled by CPER. **c-f.** Huh7-ACE2 cells (**c**, **d**) or Vero-TMPRSS2 cells (**e**, **f**) were infected with SARS-CoV-2 WT (Nluc-2A-ORF6) or ΔORF6 and supernatants were collected at 6, 12 and 24 h post infection. Viral RNA in the supernatants was quantified using qRT-PCR (**c**, **e**), and the viral titer was quantified using plaque forming assay (**d**, **f**). Two-tailed Student's t-test, n.s.: not significant. error bars represent SD (n=3).

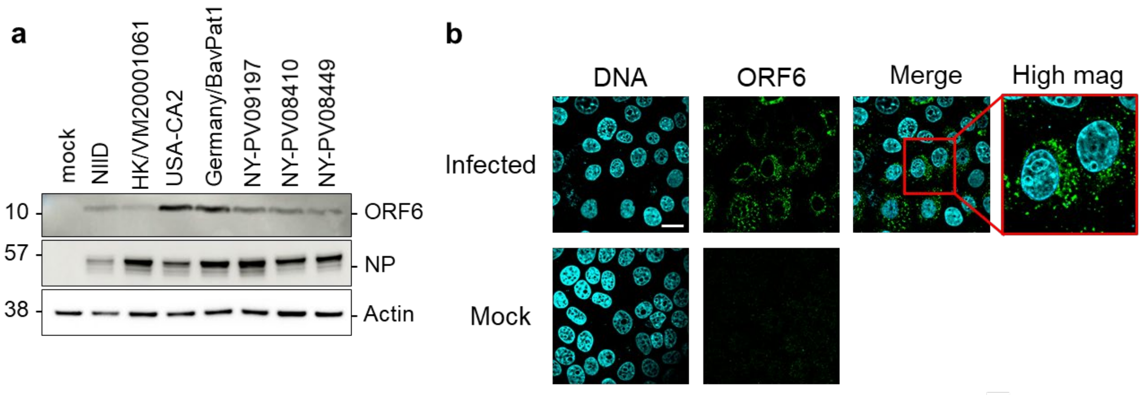


**Supplementary Figure 8. Identification of ORF6 in different SARS-CoV-2 strains by a newly established antibody**

**a)** VeroE6/TMPRSS2 cells were infected with several SARS-CoV-2 strains, and cell lysates were collected for detection of the ORF6 protein using western blotting. NP and Actin were used as an infection control and internal control, respectively. **b)** Immunofluorescence of ORF6 in VeroE6/TMPRSS2 cells infected with or without SARS-CoV-2 from NIID. The squares with a red line show a magnified marge image. Scale bars: 20 µm.

**Supplemental Table 1. Oligo sequences for plasmid constructions**

| | Name | Sequence | Vectors |
|---|---|---|---|
| Mammalian expression vector | ORF6_Fw | 5'-GCTGTACAAGGAATTCATGTTTCATCTCGTTGACTTTCAGG-3' | pCAG AcGFP-HA |
| | ORF6_Rv | 5'-GGTATCCTCTGAATTCATCAATCTCCATTGGTTGCTC-3' | |
| | ORF6-M1_Fw | 5'-GCTGTACAAGGAATTCATGTTTCATCTCGTTGACTTTCAGG-3' | |
| | ORF6-M1_Rv | 5'-GGTATCCTCTGAATTCATCAATCTCCATTGGTTGCTCTTCATC TGCTGCTGCTGCTTTATTCTCAGTTAGTGACT-3' | |
| | ORF6-M2_Fw | 5'-GCTGTACAAGGAATTCATGTTTCATCTCGTTGACTTTCAGG-3' | |
| | ORF6-M2_Rv | 5'-GGTATCCTCTGAATTCATCAATCTCCATTGGTTGTGCTGCTG CTAATTGAGAATATTTATTCT-3' | |
| | ORF6-M3_Fw | 5'-GCTGTACAAGGAATTCATGTTTCATCTCGTTGACTTTCAGG-3' | |
| | ORF6-M3_Rv | 5'-GGTATCCTCTGAATTCTGCTGCTGCTGCTGCCTCTTCA TCTAATTGAGAAT-3' | |
| | ORF6 Δ9-N_Fw | 5'-GCTGTACAAGGAATTCATGTTTCATCTCGTTGACTTTCAGG-3' | |
| | ORF6 Δ9-N_Rv | 5'-ATGATGTAAGTCCTCATAATAATTAGTAATATC-3' | |
| | ORF6 Δ9-C_Fw | 5'-TGAGGACTTACATCATAAACCTCATAATTAAAA-3' | |
| | ORF6 Δ9-C_Rv | 5'-GGTATCCTCTGAATTCATCAATCTCCATTGGTTGCTC-3' | |
| | STAT1_Fw | 5'-CGATGACAAGGGATCCATGTCTCAGTGGTACGAAC-3' | pcDNA5/FRT/3x FLAG |
| | STAT1_Rv | 5'-GGCTCTCGCTGAATTCCTATACTGTGTTCATCATACT-3' | |
| | SV40T-NLS_Fw | 5'-GATCTCCTCCAAAAAAGAAGAGAAAGGTAGAAGACG-3' | mCherry-C1 |
| | SV40T-NLS_Rv | 5'-TCGACGTCTTCTACCTTTCTCTTCTTTTTTGGAGGA-3' | |
| Bacteria expression | ORF6_Fw | 5'- GACGATATCAGGATCCATGTTTCATCTCGTTGACTTTCAGG-3' | pGEX6P2-GFP |
| | ORF6_Rv | 5'- GATGCGGCCGCTCGAGATCAATCTCCATTGGTTGCTC-3' | |
| | M0_Fw | 5'-GATCCGAATTCTATTCTCAATTAGATGAAGAGCAACCAATGG AGATTGATCCC-3' | pGEX2T-GFP |
| | M0_Rv | 5'-GGGATCAATCTCCATTGGTTGCTCTTCATCTAATTGAGAATAG AATTCG-3' | |
| | M1_Fw | 5'-GATCCGAATTCGCAGCAGCAGCAGATGAAGAGCAACCAATGG AGATTGATCCC-3' | |
| | M1_Rv | 5'-GGGATCAATCTCCATTGGTTGCTCTTCATCTGCTGCTGCTGC GAATTCG-3' | |
| | M2_Fw | 5'-GATCCGAATTCTATTCTCAATTAGCAGCAGCACAACCAATGGA GATTGATCCC-3' | |
| | M2_Rv | 5'-GGGATCAATCTCCATTGGTTGTGCTGCTGCTAATTGAGAATA GAATTCG-3' | |
| | M3_Fw | 5'-GATCCGAATTCTATTCTCAATTAGATGAAGAGGCAGCAGCAG CAGCAGCACCC -3' | |
| | M3_Rv | 5'-GGGTGCTGCTGCTGCTGCTGCCTCTTCATCTAATTGAGAATA GAATTCG-3' | |

**Supplemental Table 2. Primers for qRT-PCR**

| Name | Sequence |
|---|---|
| IP-10_Fw | 5’-GGCCATCAAGAATTTACTGAAAGCA-3’ |
| IP-10_Rv | 5’-TCTGTGTG GTCCATCCTTGGAA-3’ |
| β-actin for IP-10_Fw | 5'-TTCCAGGAGCGAGATCCCT-3' |
| β-actin for IP-10_Rv | 5'-CACCCATGACGAACATGGG-3' |
| SARS-CoV-2_N2_Fw | 5’-AAATTTTGGGGACCAGGAAC-3’ |
| SARS-CoV-2_N2_Rv | 5’-TGGCAGCTGTGTAGGTCAAC-3’ |
| β-actin for N2_Fw | 5’-TTGCTGACAGGATGCAGAAG-3’ |
| β-actin for N2_Rv | 5’-GTACTTGCGCTCAGGAGGAG- 3’ |

**Supplemental Table 3. Primers for CPER reaction**

| | |
|---|---|
| CPER#1-Fw | 5'-GAGCTCGTTTAGTGAACCGTATTAAAGGTTTATACCTTCC-3' |
| CPER#1-Rv | 5'-CTATCACAGTGTCATCACCAAAAGTAACCTTTGTTGGTGC-3' |
| CPER#2-Fw | 5'-CCTTCACACTCAAAGGCGGTGCACCAACAAAGGTTACTTT-3' |
| CPER#2-Rv | 5'-CTTCTACACCCTTAAGGGTTGTCTGCTGTTGTCCACAAGT-3' |
| CPER#3-Fw | 5'-TCTTGAACGTGGTGTGTAAAACTTGTGGACAACAGCAGAC-3' |
| CPER#3-Rv | 5'-TAACTTTAATTAACTGCTTCAACCAATTATTAACAATTTT-3' |
| CPER#4-Fw | 5'-AGATAGCACTTAAGGGTGGTAAAATTGTTAATAATTGGTT-3' |
| CPER#4-Rv | 5'-GAACCCTTAATAGTGAAATTGGGCCTCATAGCACATTGGT-3' |
| CPER#5-Fw | 5'-TGGTTCACCATCTGGTGTTTACCAATGTGCTATGAGGCCC-3' |
| CPER#5-Rv | 5'-TTTATGTCTACAGCACCCTGCATGGAAAGCAAAACAGAAA-3' |
| CPER#6-Fw | 5'-TCACTACTTTCTGTTTTGCTTTCCATGCAGGGTGCTGTAG-3' |
| CPER#6-Rv | 5'-ACCAGAAGCAGCGTGCATAGCAGGGTCAGCAGCATACACA-3' |
| CPER#7-Fw | 5'-CTTAGTTTTAAGGAATTACTTGTGTATGCTGCTGACCCTG-3' |
| CPER#7-Rv | 5'-TGGGTAAGCATCTATAGCTAAAGACACGAACCGTTCAATC-3' |
| CPER#8-Fw | 5'-CAGATGGTACACTTATGATTGAACGGTTCGTGTCTTTAGC-3' |
| CPER#8-Rv | 5'-AGGTGTGTAGGTGCCTGTGTAGGATGTAACCCAGTGATTA-3' |
| CPER#9-Fw | 5'-AAGATTGTAGTAAGGTAATCACTGGGTTACATCCTACACA-3' |
| CPER#9-Rv | 5'-GACTAGAGACTAGTGGCAATAAAACAAGAAAAACAAACAT-3' |
| CPER#10-Fw | 5'-TGTTAACAACTAAACGAACAATGTTTGTTTTTCTTGTTTT-3' |
| CPER#10-Rv | 5'-CAAATGAGGTCTCTAGCAGCAATATCACCAAGGCAATCAC-3' |
| CPER#11-Fw | 5'-GCTTCATCAAACAATATGGTGATTGCCTTGGTGATATTGC-3' |
| CPER#11-Rv | 5'-TAAGCTCTTCAACGGTAATAGTACCGTTGGAATCTGCCAT-3' |
| CPER#12-Fw | 5'-TTGGAACTTTAATTTTAGCCATGGCAGATTCCAACGGTAC-3' |
| CPER#12-Rv | 5'-TACATTCTTGGTGAAATGCAGCTACAGTTGTGATGATTCC-3' |
| CPER#13-Fw | 5'-AAATTTCTTGTTTTCTTAGGAATCATCACAACTGTAGCTG-3' |
| CPER#13-Rv | 5'-TTGTCATTCTCCTAAGAAGCTA-3' |
| CPER#14-Fw | 5'-CTATCCCCATGTGATTTTAATAGCTTCTTAGGAGAATGAC-3' |
| CPER#14-Rv | 5'-GGAAGGTATAAACCTTTAATACGGTTCACTAAACGAGCTC-3' |

Unprocessed western blot/CBB gel data

Miyamoto et al.,

The area circled by the red square is the area shown in figures.

Fig. 2f

**Fig. 2f: STAT1**

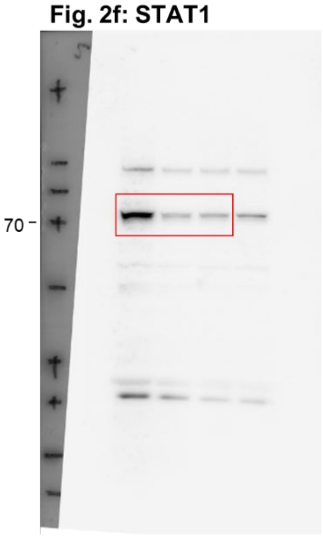


**Fig. 2f: Actin**

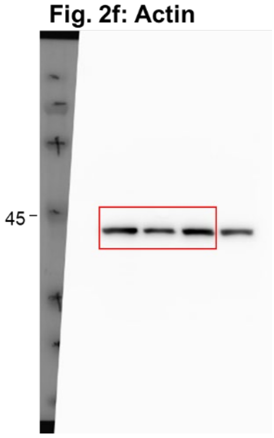


Fig. 3e

**Fig. 3e: Flag-STAT1, Light chain**

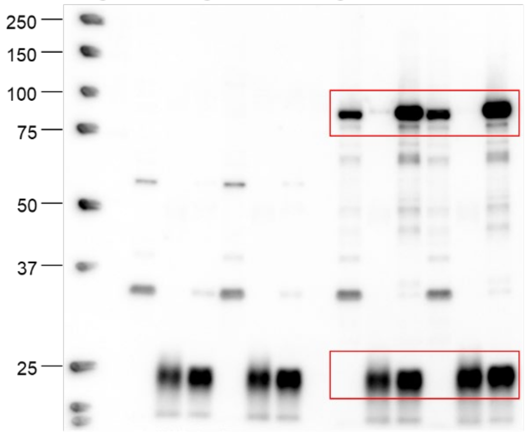


**Fig. 3e: AcGFP-ORF6**

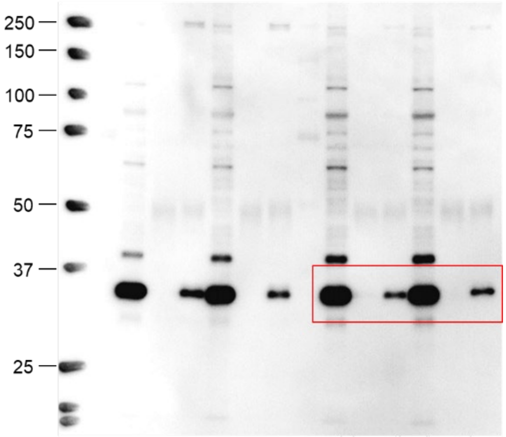


**Fig. 3e: PY-STAT1**

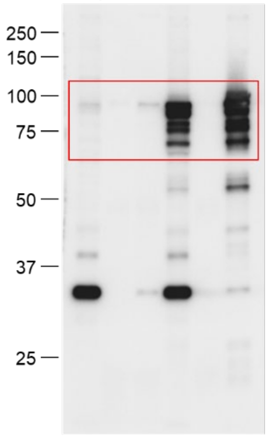

Fig. 4a,b

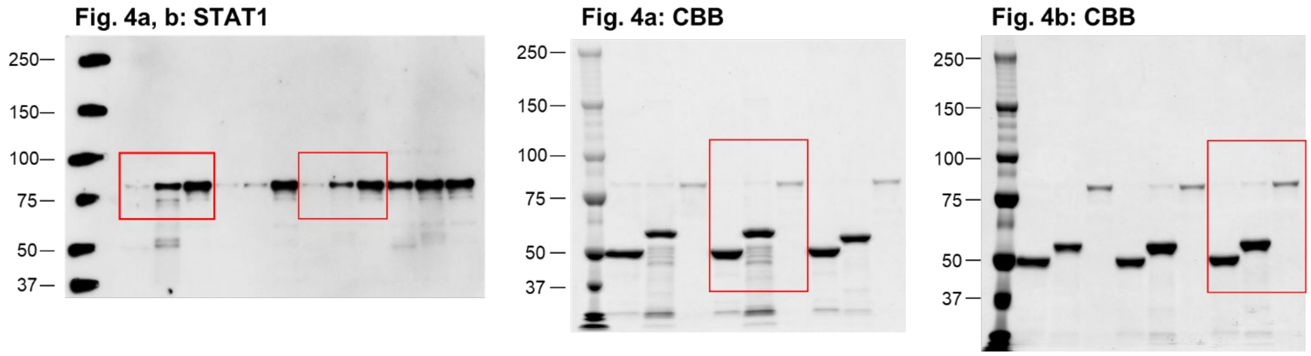


Fig. 4c

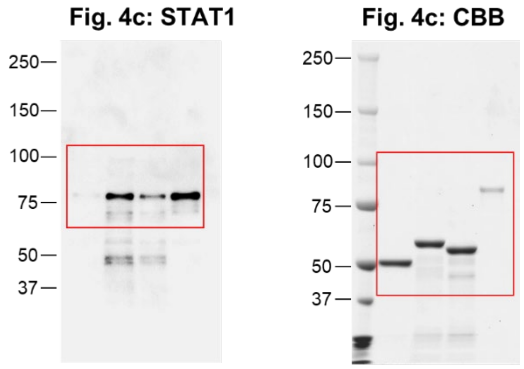


Fig. 4d

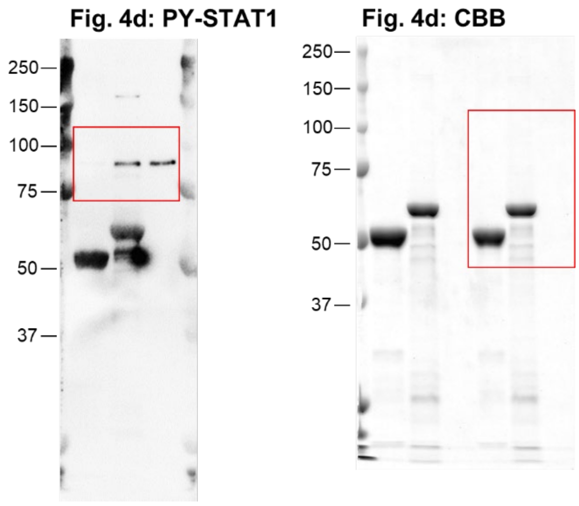

# Fig. 4e

**Fig. 4e: PY-STAT1**

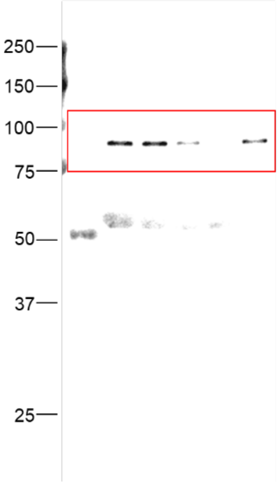


**Fig. 4e: CBB**

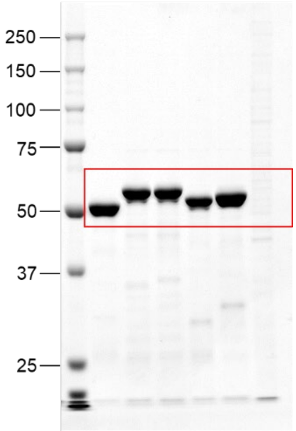


# Fig. 5c

**Fig. 5c: Impαs**

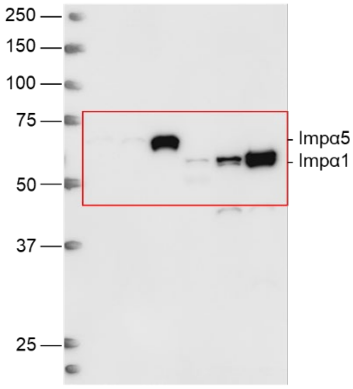


**Fig. 5c: CBB**

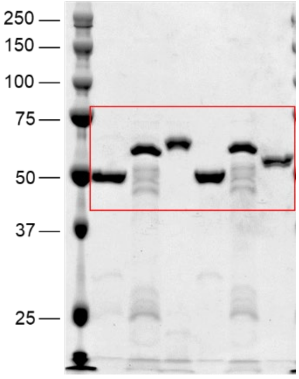


# Fig. 5d

**Fig. 5d: Impα1**

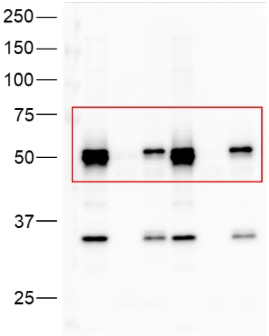


**Fig. 5d: Impα5**

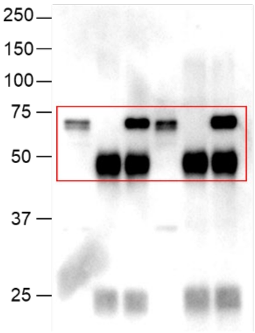


**Fig. 5d:**
**Impα5/AcGFP-ORF6 Impα1/AcGFP-ORF6**

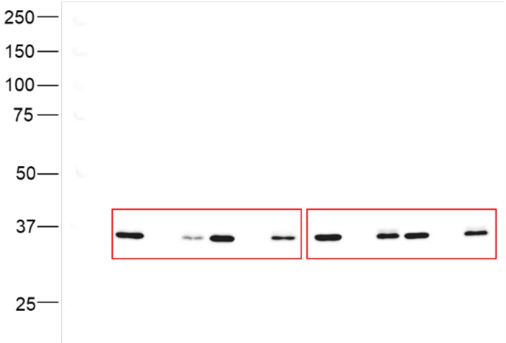

Fig. 5d

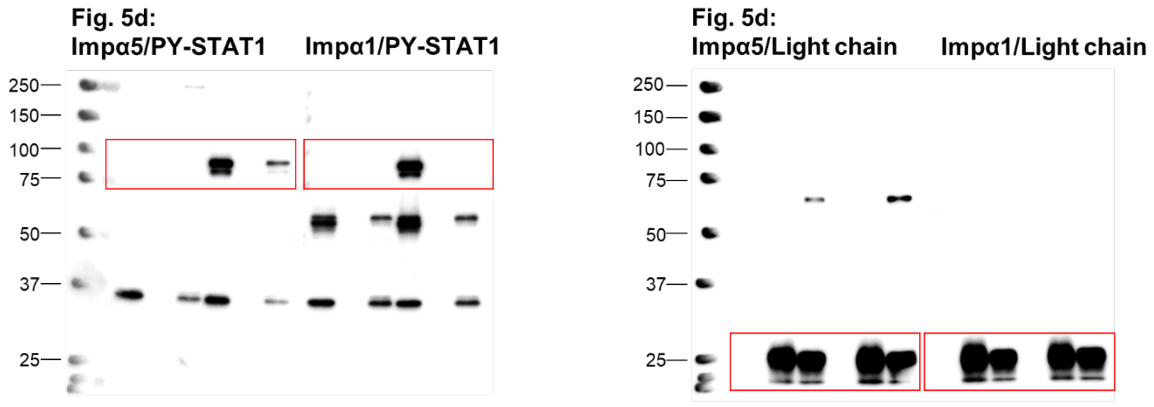


Fig. 6c

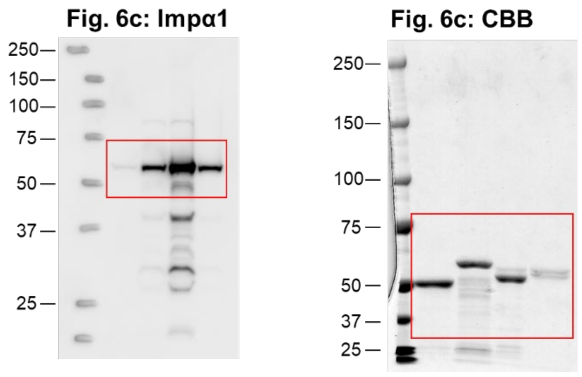


Fig. 7a

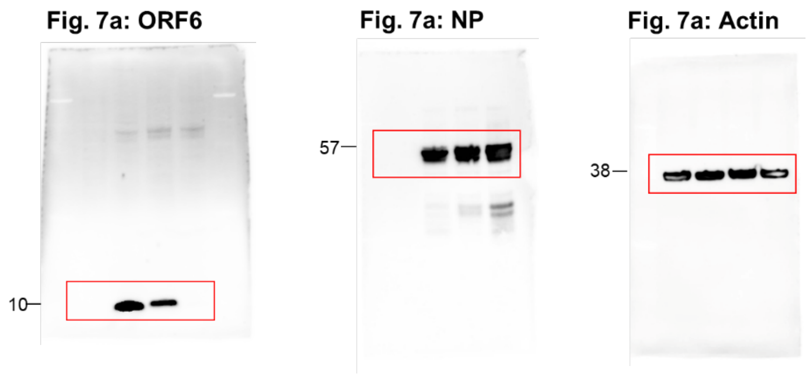

Suplemental Fig. 8a

**Supple Fig. 8a: ORF6**

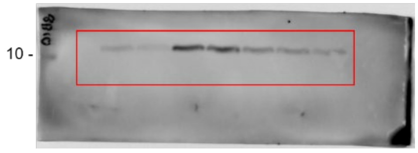


**Supple Fig. 8a: NP**

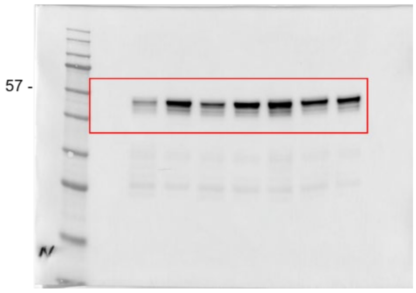


**Supple Fig. 8a: Actin**

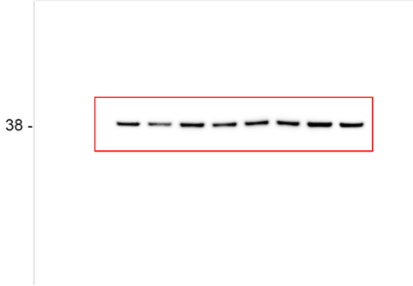

Supplement: Supplementary file 2 — Supplimental information [file 42003_2022_3427_MOESM2_ESM.pdf]
