# Supplementary material for: SARS-CoV-2 ORF6 disrupts nucleocytoplasmic trafficking to advance viral replication
Source: Commun Biol. 2022 May 19;5:483. doi: 10.1038/s42003-022-03427-4 (PMC9120032; doi:10.1038/s42003-022-03427-4)
Supplement: Supplementary file 3 — Description of Additional Supplementary Files [file 42003_2022_3427_MOESM3_ESM.pdf]

Description of Additional Supplementary Files

File name: Supplementary Data 1
Description: The source data behind the graphs in the main figures.
