# Supplementary material for: SARS-CoV-2 ORF6 disrupts nucleocytoplasmic trafficking to advance viral replication
Source: Commun Biol. 2022 May 19;5:483. doi: 10.1038/s42003-022-03427-4 (PMC9120032; doi:10.1038/s42003-022-03427-4)
Supplement: Supplementary file 5 — Reporting summary [file 42003_2022_3427_MOESM5_ESM.pdf]

nature research

Corresponding author(s): Yoichi Miyamoto

Last updated by author(s): Apr 15, 2022

Reporting Summary

Nature Research wishes to improve the reproducibility of the work that we publish. This form provides structure for consistency and transparency in reporting. For further information on Nature Research policies, see our Editorial Policies and the Editorial Policy Checklist.

Statistics

For all statistical analyses, confirm that the following items are present in the figure legend, table legend, main text, or Methods section.

n/a	Confirmed	
☐	☒	The exact sample size (*n*) for each experimental group/condition, given as a discrete number and unit of measurement
☐	☒	A statement on whether measurements were taken from distinct samples or whether the same sample was measured repeatedly
☐	☒	The statistical test(s) used AND whether they are one- or two-sided<br>*Only common tests should be described solely by name; describe more complex techniques in the Methods section.*
☐	☒	A description of all covariates tested
☐	☒	A description of any assumptions or corrections, such as tests of normality and adjustment for multiple comparisons
☐	☒	A full description of the statistical parameters including central tendency (e.g. means) or other basic estimates (e.g. regression coefficient) AND variation (e.g. standard deviation) or associated estimates of uncertainty (e.g. confidence intervals)
☐	☒	For null hypothesis testing, the test statistic (e.g. *F*, *t*, *r*) with confidence intervals, effect sizes, degrees of freedom and *P* value noted<br>*Give P values as exact values whenever suitable.*
☒	☐	For Bayesian analysis, information on the choice of priors and Markov chain Monte Carlo settings
☒	☐	For hierarchical and complex designs, identification of the appropriate level for tests and full reporting of outcomes
☒	☐	Estimates of effect sizes (e.g. Cohen's *d*, Pearson's *r*), indicating how they were calculated

Our web collection on statistics for biologists contains articles on many of the points above.

Software and code

Policy information about availability of computer code

Data collection	No software was used to collect the data in this study.
Data analysis	Graph Pad Prism 7 (GraphPad Software) and Excel (Microsoft).

For manuscripts utilizing custom algorithms or software that are central to the research but not yet described in published literature, software must be made available to editors and reviewers. We strongly encourage code deposition in a community repository (e.g. GitHub). See the Nature Research guidelines for submitting code & software for further information.

Data

Policy information about availability of data

All manuscripts must include a data availability statement. This statement should provide the following information, where applicable:

- Accession codes, unique identifiers, or web links for publicly available datasets
- A list of figures that have associated raw data
- A description of any restrictions on data availability

n/a

Field-specific reporting

Please select the one below that is the best fit for your research. If you are not sure, read the appropriate sections before making your selection.

☒ Life sciences ☐ Behavioural & social sciences ☐ Ecological, evolutionary & environmental sciences

For a reference copy of the document with all sections, see nature.com/documents/nr-reporting-summary-flat.pdf

Life sciences study design

All studies must disclose on these points even when the disclosure is negative.

Sample size	No statistical methods were used to predetermine sample size, and did not perform sample-size calculation.
Data exclusions	We did not exclude any samples from the analysis.
Replication	Experiments were repeated and our data are based on at least two to four independent experiments with similar results.
Randomization	Age matched animals were randomly allocated into experimental groups.
Blinding	Investigators were not blinded to group allocation.

Reporting for specific materials, systems and methods

We require information from authors about some types of materials, experimental systems and methods used in many studies. Here, indicate whether each material, system or method listed is relevant to your study. If you are not sure if a list item applies to your research, read the appropriate section before selecting a response.

Materials & experimental systems

n/a	Involved in the study	
☐	☒	Antibodies
☐	☒	Eukaryotic cell lines
☒	☐	Palaeontology and archaeology
☐	☒	Animals and other organisms
☒	☐	Human research participants
☒	☐	Clinical data
☒	☐	Dual use research of concern

Methods

n/a	Involved in the study	
☒	☐	ChIP-seq
☒	☐	Flow cytometry
☒	☐	MRI-based neuroimaging

Antibodies

Antibodies used

The following primary antibodies were used in the present study: Phospho-STAT1 (Tyr701) (#9167 [58D6], Cell Signaling Technology (CST) Inc., Danvers, MA, USA), STAT1 (#9172, CST), importin α1/KPNA2 (ab84440, Abcam, Cambridge, MA, USA; #610486, Anti-Karyopherin α (Rch1), BD Transduction Lab., San Jose, CA, USA), HIF-1α (ab51608 [EP1215Y], Abcam), NF-κB p65 (#8242 [D14E12], CST), importin β1 (ab2811 [3E9], Abcam), CAS (ab96755, Abcam), Lamin A/C (sc-6215, Santa Cruz, Dallas, TX, USA), Flag (M2 [F1804], Sigma-Aldrich), GFP (A-11122, rabbit, Thermo Fisher Scientific, Waltham, MA, USA), GFP (M048-3, mouse, MBL, Nagoya, Japan), NP (3A9, mouse mAb, Cell Engineering Co., Osaka, Japan), Actin (A2228, Sigma-Aldrich), and HA (MMS-101R, Biolegend, San Diego, CA, USA).

Horseradish peroxidase (HRP)-conjugated anti-rabbit (#111-035-003), anti-mouse (#115-035-003), or anti-rat (#112-035-003) secondary antibodies (Jackson ImmunoResearch Inc. West Grove, PA, USA) were used for western blotting. The secondary antibodies used for indirect immunofluorescence were as follows: Alexa Fluor Plus 488 conjugated anti-rabbit (A32731) or anti-mouse (A32723), and Alexa Fluor 594 conjugated anti-rabbit (A21207) or anti-mouse (A21203) (Invitrogen).

Experimental procedures for production of monoclonal antibody were approved by the CEC Animal Care and Use Committee (permission number: CMJ-044) and performed according to CEC Animal Experimentation Regulations. A rat monoclonal antibody that specifically recognized the SARS-CoV-2 ORF6 protein was generated using the rat medial iliac lymph node method (ref.44). An 8-week-old female WKY rat was injected with 100 μL of emulsions containing ORF6 peptide (CEEQPMEID)-conjugated KLH and Freund's complete adjuvant into the rear footpads. Seventeen days after the first immunization, an additional immunization of SARS-CoV-2 ORF6 peptide-KLH was administered without an adjuvant into the tail base of the rat. Four days after the second immunization, cells from the iliac lymph nodes of the immunized rat were fused with mouse myeloma Sp2/0-Ag14 cells at a ratio of 5:1 in 50% polyethylene glycol. The resulting hybridoma cells were plated onto 96-well plates and cultured in HAT selection medium (Hybridoma-SFM [Life Technologies, Grand Island, CA, USA]; 10% FBS; 1 ng/mL mouse IL-6; 100 μM hypoxanthine [Sigma-Aldrich, St. Louis, MO, USA]; 0.4 μM aminopterin [Sigma-Aldrich]; and 16 μM thymidine [WAKO, Osaka, Japan]). The SARS-CoV-2 ORF6-specific antibody was screened using ELISA, western blotting, and immunostaining of hybridoma supernatants. Finally, hybridoma clone producing the monoclonal antibody, later named 8B10, was selected. Using a rat isotyping kit the MAb 8B10 was found to be an IgG

	1 (k) antibody subtype. The monoclonal antibody against SARS-CoV-2 NP (3A9 clone) was generated by Cell Engineering Corporation (Osaka, Japan). Western blotting for the protein in cells infected with different viral strains, which were obtained from the National Institute of Infectious Diseases (NIID) in Japan, Hong Kong (HK)/VM20001061, USA-CA2, Germany/BavPat1, New York (NY)-PV09197, NY-PV08410, and NY-PV08449 (Supplementary Fig. 8a). Indirect immunofluorescence images for the SARS-CoV-2-infected VeroE6/TMPRSS2 cells, which is consistent with the previous observation in SARS-CoV-infected Vero E6 cells (ref.45), are represented in Supplementary Fig. 8b.
Validation	All antibodies validation are available on the manufacturers' websites except for the anti-ORF6 antibody which was validated in this manuscript.

Eukaryotic cell lines

Policy information about cell lines

Cell line source(s)	HeLa cells (ATCC), HEK293 cells (NIBIOHN), Huh7 cells (National institute of infectious diseases in Japan.), Huh7-ACE2 which were generated by infection with lentivirus expressing human ACE2, VeroE6/TMPRSS2 cells (NIBIOHN, JCRB1819), and Vero E6 replicon stable cells (ref. 51)
Authentication	All cell lines were not authenticated.
Mycoplasma contamination	All cell lines were clarified no mycoplasma contamination.
Commonly misidentified lines (See ICLAC register)	No commonly misidentified cell lines were used.

Animals and other organisms

Policy information about studies involving animals; ARRIVE guidelines recommended for reporting animal research

Laboratory animals	All animal experiments using the SARS-CoV-2 virus were performed in biosafety level 3 (ABSL3) facilities at the Research Institute for Microbial Diseases, Osaka University. The animal experiments and the study protocol were approved by the Institutional Committee of Laboratory Animal Experimentation of the Research Institute for Microbial Diseases, Osaka University (R02-08-0). Throughout the study, we focused on minimizing animal suffering and reducing the number of animals used in the experiments. Four week-old male Syrian hamsters were purchased from SLC (Shizuoka, Japan).
Wild animals	n/a
Field-collected samples	n/a
Ethics oversight	The animal experiments using Syrian hamsters were approved by the Institutional Committee of Laboratory Animal Experimentation of the Research Institute for Microbial Diseases, Osaka University (R02-08-0). Experimental procedures in production of monoclonal antibody were approved by the CEC Animal Care and Use Committee (permission number: CMJ-044) and performed according to CEC Animal Experimentation Regulations.

Note that full information on the approval of the study protocol must also be provided in the manuscript.
